# Factors associated with disclosing men who have sex with men (MSM) sexual behaviors and HIV-positive status: A study based on a social network analysis in Nanjing, China

**DOI:** 10.1371/journal.pone.0196116

**Published:** 2018-04-19

**Authors:** Lusi Chen, Dashuai Lian, Bei Wang

**Affiliations:** Department of Epidemiology and Health statistics, Key Laboratory of Environmental Medicine Engineering, Ministry of Education, School of Public Health, Southeast University, Nanjing, Jiangsu, China; Pontificia Universidade Catolica do Rio Grande do Sul, BRAZIL

## Abstract

**Objective:**

We explored the factors associated with disclosure of men who have with sex with men (MSM) behaviors and HIV-positive status among HIV-positive MSM in Nanjing, China.

**Methods:**

Social network analysis and epidemiological methods were combined in this pilot study. Information about participants’ (egos’) characteristics and behaviors and their social network members (alters) were collected through interview-administered questionnaires. General estimating equation logistic regression analysis was applied in both univariate and multivariate analysis.

**Results:**

Eighty-seven HIV-positive MSM participated. Their mean age was 35.9 ±13.81years. They were more likely to disclose their MSM behavior to their friends [adjust Odds Ratio (AOR) = 6.43, 95% confidence interval (CI):3.08–13.42] or to the social network members who were not heterosexual [AOR = 4.40, 95%CI: 2.17–8.91]. Being participants’ friends [AOR = 5.16, 95%CI: 2.03–13.10] or family members [AOR = 6.22, 95%CI: 2.52–15.33] was significantly associated with HIV-positive status disclosure.

**Conclusion:**

HIV-positive MSM tended to disclose their MSM behaviors and HIV positive status to close friends, family members or other individuals who were HIV-positive, engaging in MSM behavior, or both. Consequently, it will be an effective way to implement HIV prevention and intervention strategies in both MSM population and their trusted social networks.

## Introduction

Human immunodeficiency virus (HIV) is the cause of acquired immunodeficiency syndrome (AIDS), which is still a severe global epidemic, especially among men who have sex with men (MSM) population. MSM contribute to an elevated HIV infection rate[1] globally due to HIV transmission through condom-less anal intercourse. The estimated HIV prevalence among MSM population ranged from 3.0–25.4% around the world [2]. In China, the predominant mode of HIV transmission has shifted from injection drug users to sexual transmission, particularly among homosexual men. From 2014 to 2017, newly HIV-infected MSM comprised over a quarter of new reported infections each year [3–5]. With the remarkable expansion of Highly Active Anti-Retroviral Therapy over the past two decades, 36.7 million people globally were living with HIV/AIDS (PLWHAs) in the end of 2016[6]. China has implemented the World Health Organization’s (WHO) guidelines[2, 7] for HIV treatment. As of 2016, over 400 thousands HIV/AIDS patients were under HIV treatment [8]. Chinese MSM can receive free HIV treatment once they are diagnosed with HIV infection, thus improving their overall health status. This population is much healthier, living longer and more sexually active then before, which may be a potential risk of HIV transmission[9]. Therefore, HIV prevention and intervention among HIV-positive MSM is critical and necessities more attention.

Disclosure of MSM behaviors and HIV-positive status can be beneficial for HIV prevention and intervention as well as MSM’s personal health. After disclosing to families or friends, MSM could receive more emotional or tangible support, increased self-esteem and decreased depression or anxiety [10]. After disclosing to sexual partners, MSM tend to have safer sex [11, 12] which may reduce HIV transmission. In addition, disclosure to health providers is related to adherence to health-care and service[13, 14], thus controlling disease progression. However, disclosure may also lead MSM to experience stigma[15], discrimination and isolation [16], as well as homophobia[17]. In China, disclosure is influenced by social and culture factors, rather than a simple personal issue. Unlike the some other countries, homosexuality is not widely accepted in Chinese mainstream culture and tradition; being gay means marginalization by others[18]. Therefore, Chinese MSM may hide their same-sex behaviors or HIV status. Furthermore, because of family values [19] including familial pressure to have offspring [20], some MSM had to marry women and engage in both homosexual and heterosexual activities. This may result in MSM transmitting HIV between homosexual and heterosexual populations.

Studies [21–23]on disclosure of same sex behavior and HIV status among Chinese MSM populations have often been conducted from the perspective of individual’s behaviors or characteristics. However, people’s behaviors and health are interconnected [24] in a social syndemics framework[25] related to their mental and physical well-being. Social network analysis provides a set of theories, techniques, and tools useful for understanding how human behavior changes as people interact with others [26]. Specifically, egocentric social network focuses on index people (egos) and how their network members (alters) affect egos. It is an appropriate way to generate detailed information from high-risk HIV populations, such as MSMs and Inject drug users. Therefore, in this pilot study, factors of disclosure of MSM behavior and HIV-positive status were investigated by social network analysis, which can provide ideas and the foundation for HIV prevention and intervention among MSM in China.

## Methods and measures

### Cross-sectional procedures and ethical approval

This pilot study was a cross-sectional study launched during March 2016 in the infectious disease clinic of Nanjing Infectious Hospital, Nanjing, China. This hospital was the designated hospital in Nanjing providing ART medication to HIV/AIDS patients. We recruited participants by convenience sampling according to the inclusion criteria (1) aged 18 years or older, (2) self-identified as a male, (3) reported having sex with men, (4) diagnosed and confirmed as HIV-infected or AIDS by Center of Disease Control or hospitals, (5) agreed to participate in the study. Medical staff primarily screened patients using inclusion criteria; then, well-trained data collectors confirmed participants were eligible. Eighty-nine MSM HIV-infected patients were approached. Eighty-seven participants were eligible and recruited into this study while two did not have time to participate. Demographic and behavioral data as well as their social network members’ information were collected from enrolled participants by using interview-administered questionnaires including egocentric social network inventory.

This was an anonymous study and each participant was reimbursed 50 CNY (Chinese Yuan) for the study. (average monthly income in Nanjing is about 2590 CNY (387U.S.Dallar)[27]). Verbal or written consent were obtained from all participants. The IEC (Institutional ethical committee) for Clinical Research of Zhongda Hospital, affiliated to Southeast University approved the ethical protocol.

### Index participants’ characteristics and sexual behaviors

All index participants completed the face-to-face interviews. Age, education, annual income, marital status, self-reported sexual orientation, and residence were measured for the demographic information. Regarding residence, participants were classified as local residents and non-local residents (migrants) according to the self-reported official household registration. Regarding sexual behaviors portion, information about having anal sex with a man or vaginal sex with a woman, number of sexual partners, type of sexual partners, condom use with sex, drug abuse and sexual transmitted infection (STIs: chlamydia, gonorrhea, genital herpes, HPV, syphilis) were collected. We defined three types of sexual partners in this study. Commercial sexual partners were those providing sexual services in economic form or providing sexual services for financial gain; casual sexual partners were non-commercial non-fixed partners, not involved in a love relationship; steady sexual partners were legal spouses and sexual partners engaging in a heterosexual or homosexual relationship as a couple.

### Egocentric social network inventory

Every index participant was required to nominate up to eight people by asking a name-generating question that who were closed (e.g. providing emotional, material, or financial support) to you during last 6 months as the social network members. Then, index participants were asked several questions about social network members’ attributes including age, gender, relationship to index participants, education, sexual orientation, HIV and STIs infection status, drug abuse, level of relationship degree with egos (on scale from 1 to 4, with 1 as best and 4 as wrost), awareness of same-sex behaviors or HIV status of index participants, and attitudes toward MSM behaviors.

Furthermore, participants were asked questions about whether there were changes in their social or sexual networks after they acquired HIV. The changes could include, but not limited to job-hopping, migration, reducing social or sexual activities, obtaining new friends, and so on. There was overlap of social and sexual networks based on whether the participants specified any members of the social networks who were also sex partners.

### Statistical analysis

Same-sex behaviors disclosure and HIV-positive status disclosure were the two outcome variables. The former was assessed by asking participants “Among your social network list, who knows you have sex with men?” The latter was assessed by asking participants “Who on your social network list have you disclosed your HIV status to?”

Logistic regression models with generalized estimating equations (GEE) were applied to explore the associations between outcome variables and attributes of index participants and their egocentric social network members. Variables correlated with outcomes in the univariate models (P<0.20) were included in the multivariable model, considering potential confounding factors. Multicollinearity of independent variables was examined by variance inflation factor (VIF). GEE is a useful approach to address repeated measures or multilevel/clustered data. We chose GEE here because each index participant and his network members could be treated as a cluster. Robust standard errors were used for estimation of 95% CI (confidence intervals). Data were collected through EgoNet™ (developed by Medical Decision Logic, Inc) and analyses were conducted using Stata Version 12 (StataCorp LLC, 4905 Lakeway Drive, College Station, TX USA 77845).

## Results

### Social demographic characteristics and risk behaviors of index participants

Our sample consisted of 87 HIV/AIDS male patients in our study. Participants’ mean age was 35.9 ±13.81years and the mean age of participants’ first MSM experience was 24.05±9.87 years. More than half (58.5%) were highly educated (i.e. college or more). Most (70.9%) were self-identified as homosexual and 21.8% were bisexual. Further, 47.1% participants had anal sex with men during last six months and 8.1% participants who had vaginal sex with women. Most reported they used a condom every time. Participants’ STIs infection ratio was 16.9%, including syphilis (9.2%), condyloma acuminate (4.6%), and herpes genitalis (1.1%). In terms of MSM behaviors and HIV-positive status disclosure, most of them disclosed to at least one of their social network members, 69.0% and 66.7% respectively. (Table 1)

**Table 1:** Demographic characteristics and risk behaviors of HIV/AIDS MSMs (n = 87), Nanjing China.

		N	Frequency%
Age		35.87±13.812	-
Education	High school or less	36	41.4
	College or more	51	58.6
	<12000	14	16.1
Annual Income (CNY)	12000–36000	14	16.1
	36000–60000	25	28.7
	60000–96000	17	19.5
	>96000	17	19.5
Marriage	Unmarried	56	64.4
	Married	18	20.7
	Divorced/widowed	13	14.9
Sexual orientation	heterosexual	6	6.9
	homosexual	61	70.1
	bisexual	19	21.8
	unknown	1	1.1
Residence	Local	37	42.5
	No-local	50	57.5
Mean network size		3.49±2.15	-
MSM disclosure		60	69.0
HIVdisclosure		58	66.7
First MSM experience age		24.05±9.87	(14–66)
Sexual role	Insertive (1)	22	27.2
	Receptive (0)	28	32.2
	Both (0.5)	31	35.5
Anal sex with men in last six months		41	47.1
	Casual sexual partners	14	16.1
	Fixed sexual partners	32	36.8
Condom using for homosexual	Never	3	7.3
	Sometimes	5	12.2
	Always	33	37.9
Heterosexual in last six months		7	8.1
	Casual sexual partners	1	1.1
	Fixed sexual partners	6	7.0
Drug use		8	9.2
STDs		14	16.9
	syphilis	8	9.2
	condyloma acuminata	4	4.6
	herpes genitals	1	1.1

### Characteristics of social network members

Three-hundred nine social network members were nominated in this study. The MSM’s average network size was 3.49±2.15 (range 0–8). Four main types of relationship were collected: family members (41.10%), steady sexual partners (5.18%), friends (32.04%), and schoolmates or colleagues (21.68%). Most had a good or intimate relationship with index participants (92.28%). Further, 72.49% of social network members were heterosexual reported by index participants. Most of them were supportive or neutral to index participants’ MSM behavior. (Table 2)

**Table 2:** Social network members’ characteristics.

		N	Frequency%
Male in social network		176	56.96
Mean network size		3.49±2.15	(0,8)
Relationship	Family members	127	41.10
	Steady homosexual partners	16	5.18
	Friends	99	32.04
	Schoolmates/colleagues	67	21.68
Age		37.3±15.23	(11,83)
Education	High school or less	142	45.95
	College or more	167	54.05
Relationship degree	Intimate	202	65.37
	Good	81	26.21
	General	26	8.41
Sexual orientation	Heterosexual	224	72.49
	Others	85	27.51
Attitude	supportive	80	25.89
	neutral	47	15.21
	opposed	8	2.59
	unknown	174	56.31
Drug use		1	0.32
STIs		1	0.32
HIV/AIDS		12	3.88

### MSM behavior disclosure and HIV-positive status disclosure

Less than half of the social network members knew the participants’ same-sexual behavior (43.69%) or HIV-positive status (35.92%). Table 3 shows the results of univariate and multivariate analysis on factors associated with MSM behavior and HIV-positive status disclosure. VIF ranged from 1.03 to 1.26, which indicated no multicollinearity among these variables. From the univariate analysis of MSM disclosure, we found that participants who had their first homosexual experience at an older age (OR = 0.96, 95%CI 0.92–1.00), or who did not have anal sex in last 6 months (OR = 0.43, 95%CI 0.21–0.89) were less likely to disclose their same-sex behavior.

**Table 3:** GEE logistic regression model of factors associated with MSM behavior disclosure and HIV-positive status disclosure.

			MSM behavior disclosure			HIV-positive disclosure		
characteristics	*COR*	*95%CI*	*AOR*	*95%CI*	*COR*	*95%CI*	*AOR*	*95%CI*
***Ego***								
Age	0.99	(0.96,1.02)	-	-	1.00	(0.98,1.03)	-	-
Education	0.82	(0.40,1.69)	-	-	0.53	(0.26,1.10)	-	-
Income	1.05	(0.81,1.37)	-	-	0.97	(0.74,1.27)	-	-
Marriage	0.88	(0.59,1.32)	-	-	1.04	(0.69,1.56)	-	-
Sex orientation	0.50	(0.25,1.02)	-	-	0.85	(0.43,1.71)	-	-
residence	1.82	(0.88,3.76)	-	-	1.68	(0.80,3.55)	2.48*	(1.04,5.92)
Age of first MSM	0.96*	(0.92,1.00)	0.28*	(0.08,0.96)	1.00	(0.96,1.04)	-	-
Sexual role	0.79	(0.53,1.18)	-	-	0.89	(0.58,1.35)	-	-
Anal sex in last 6 months	0.43*	(0.21,0.89)	-	-	0.69	(0.33,1.45)	-	-
Change of social network after infection	0.59	(0.28,1.22)	-	-	0.27*	(0.12,0.64)	0.30*	(0.12,0.76)
Change of sexual network after infection	1.28	(0.56,2.94)	-	-	0.52	(0.21,1.29)	-	-
***Alters***								
Gender	0.80	(0.56,1.14)	-	-	1.06	(0.74,1.51)	-	-
Relationship								
Friends	7.81*	(4.20,14.54)	6.43*	(3.08,13.42)	5.95*	(2.69,13.13)	5.16*	(2.03–13.10)
Family members	1.73	(0.93,3.22)	2.35*	(1.14,4.84)	5.13*	(2.31,11.42)	6.22*	(2.52–15.33)
Education	1.64*	(1.06, 2.54)	-	-	1.02	(0.66,1.58)	-	-
Homosexual& Bisexual	5.63*	(3.52,9.01)	4.40*	(2.17,8.91)	1.62*	(1.05,2.50)	-	-
HIV status	-	-			37.66*	(4.28,331.46)	-	-

Note

* means P<0.05 and significant.

Considering network members’ characteristics, we found positive association with MSM behavior disclosure. Participants were more likely disclose to network members who were friends (OR = 7.81, 95%CI 4.20, 14.54) rather than family members, colleagues or schoolmates. If network members were not heterosexual (OR = 5.63, 95%CI 3.52–9.01) or were highly educated (OR = 1.63, 95%CI 1.06–2.54), participants tended to communicate their same-sex behaviors. Consistent results were generated in the multivariate analysis: participants’ age of first MSM experience (AOR = 0.28, 95%CI 0.08, 0.96) were negatively related to MSM disclosure. In addition, being friends (AOR = 6.43, 95%CI 3.08–13.42) or not heterosexual (AOR = 4.40, 95%CI 2.17–8.91) among network members was positively related to MSM disclosure.

The same strategy was operationalized to explore factors linked to HIV-positive status disclosure. In the univariate analysis, if participants recalled that their social network changed after infecting with HIV, they were less likely to disclose their HIV-positive status (OR = 0.27, 95% CI 0.12, 0.64). Regarding social network members’ attributes, being friends (OR = 5.95, 95%CI 2.69–13.13) or family members (OR = 5.73, 95%CI 2.31–11.42), infected with HIV (OR = 37.66, 95%CI 4.28–331.46) were positively associated with knowing participants’ HIV-positive status. The results of the multivariate analysis revealed that no-local participants were more likely to disclose their HIV-positive status (AOR = 2.48, 95%CI 1.04, 5.92) while social network changes was negatively associated with HIV-positive status disclosure (AOR = 0.03, 95%CI 0.12–0.76). Attributes of network members related with HIV status disclosure was consistent with univariate analysis: Participants’ friends (AOR = 5.16, 95%CI 2.03–13.10) or family members (AOR = 6.22, 95%CI 2.52–15.33) tended to know their HIV-positive status.

## Discussion

In this study, we analyzed 87 HIV-positive MSM and their 309 elicited network members. Results indicated that the social network size of HIV-infected MSM was relatively small compared with other MSM [28, 29]. According to studies from the U.S, the network size of African American MSM typically ranged from 5 to 8[30–32]. However, no relationship between network size and disclosure was observed in this study.

Findings from this study suggested that the MSM’s disclosure rate of both HIV-status and same-sex behavior were low. Additionally, one-third of participants never disclosed to their network members. They may worry about experiencing stigmas, isolations, misunderstanding, or stress from families, friends and colleagues after disclosing their lifestyle [33]. Another possible reason may be the cultural context of Asia, people want to protect their family from shame [19, 34, 35].

Those who had disclosed their MSM behavior and HIV status were more likely to disclose to close friends, family members, or non-heterosexual alters. This was consistent with findings from other studies: For example, a study of migrant MSM in Beijing MSM reported that most of them disclosed their same-sex behavior to friends (69%), followed by family (25%)[21]. Another similar study[23, 36] found that the main disclosure targets of HIV-infected MSM were family members, friends. It is understandable that intimate people such as family or close friends can provide comfort, encouragement or economic support which will promote their physical and mental health[21, 23, 36–38].

Additionally, some participants’ characteristics were also related to disclosure. Individuals who had their first homosexual experience at an older age were less likely to disclose their same-sex behavior. This may be because they did not have enough time to accept their sexual orientation and its related behaviors; therefore, they may withhold MSM behaviors. However, few studies have comparable results. Researches concerning same-sex behavior focused mainly on age and HIV risk [39–42], while others found that older MSM reported less MSM behaviors [32, 43].

Participants whose social network was the same as before were less likely to disclose their HIV status. Perhaps those who did not want to disclose their HIV status tried to act as if everything was normal; therefore, their social network did not change. Maybe this was a way to protect themselves from stress, stigma and discrimination. Daskalopoulou’s[44] study can support this point that compared with those who disclosed to “none” or “some” friends, MSM who disclosed to “more” or “all” of their family or friends were more likely to have symptoms of depression and anxiety. Further, non-local participants were more likely to disclose their HIV status compared to local participants. This was partially consistent with what we found above. Local participants already have established their social network; they were more likely to live as before to maintain stability. However, migrants in China have less access to healthcare[45]. Consequently, non-local residents may hope to obtain more support by disclosing their HIV.

This study had some limitations. First, it is hard to recruit HIV-positive MSM because of their specific status. We used a cross-sectional design and convenience sampling to recruit participants at Nanjing infectious hospital, which eased this process. Thus, our results may be not generalized since we employed cross-sectional design and a small sample at one site. Further studies should utilize the RDS (Random Driven sampling) at multi-sites to collected more participants. Second, self-reported egocentric network study may cause inaccuracy and bias although it was a preferable method to collect rich information[46]. Further studies should include interviews with the help of MSM-community [47] and train well skilled data collector to obtain high-quality data. Third, because we set the upper limit for social network size, there is a lack of variability of social network members. Future research should increase the upper limit to acquire more detailed information about network members’.

## Conclusion

Despite these limitations, this study contributes to the body of knowledge on social support among the MSM in Nanjing, China. As showed in our study, HIV-positive MSM in Nanjing have a small social network and a noteworthy number of them did not want to share their HIV status and sexual orientation with others. Close friends and family members were the main targets among those who did disclose these characteristics. Therefore, HIV prevention and intervention measures should be provided to both MSM and their network members, which may reduce HIV transmission and increase HIV-infected individuals’ life quality.

## Supporting information

S1 Table STROBE statement—Checklist of items that should be included in reports of observational studies. (DOCX)

S1 File DATA. (XLS)
